# Ultraviolet sensitivity of WASH (water, sanitation, and hygiene) -related helminths: A systematic review

**DOI:** 10.1371/journal.pntd.0007777

**Published:** 2019-09-19

**Authors:** Lucinda Hazell, Laura Braun, Michael R. Templeton

**Affiliations:** Department of Civil and Environmental Engineering, South Kensington Campus, Imperial College London, London, United Kingdom; IRNASA, CSIC, SPAIN

## Abstract

**Background:**

Helminthiases are a group of disabling neglected tropical diseases that affect billions of people worldwide. Current control methods use preventative chemotherapy but reinfection is common and an inter-sectoral approach is required if elimination is to be achieved. Household and community scale water treatment can be used to provide a safe alternative water supply for contact activities, reducing exposure to WASH (water, sanitation, and hygiene) -related helminths. With the introduction of ultraviolet light emitting diodes (UV-C LEDs), ultraviolet (UV) disinfection could be a realistic option for water treatment in low-income regions in the near future, to provide safe alternative water supplies for drinking and contact activities such as handwashing, bathing, and laundry, but currently there is no guidance for the use of UV or solar disinfection against helminths.

**Methodology:**

A qualitative systematic review of existing literature was carried out to establish which WASH-related helminths are more susceptible to UV disinfection and identify gaps in research to inform future studies. The search included all species that can infect humans and can be transmitted through water or wastewater. Five online databases were searched and results were categorized based on the UV source: sunlight and solar simulators, UV-A and UV-B (long wavelength) sources, and UV-C (germicidal) sources.

**Conclusions:**

There has been very little research into the UV sensitivity of helminths; only 47 studies were included in this review and the majority were carried out before the standard protocol for UV disinfection experiments was published. Only 18 species were studied; however all species could be inactivated by UV light. Fluences required to achieve a 1-log inactivation ranged from 5 mJ/cm^2^ to over 800 mJ/cm^2^. Larval forms were generally more sensitive to UV light than species which remain as an egg in the environment. This review confirms that further research is required to produce detailed recommendations for household or community scale UV-C LED or solar disinfection (SODIS) of water for preventing helminthiases.

## Author summary

Helminth infections are currently controlled by mass administration of anthelmintic drugs which are effective at treating the diseases but cannot prevent reinfection. As we work to eliminate these diseases, complimentary control methods such as improving access to water, sanitation, and hygiene will be crucial to reduce re-exposure and cut transmission. UV disinfection is a widely used form of water treatment but it is often seen as incompatible with low income regions. Recently developed UV-C LEDs and SODIS offer alternative sources of UV light that may be more suitable for this context, but there is little guidance about how we can use this technology to prevent helminth infections. We carried out a systematic review to establish which helminths are more sensitive to UV light and identify the areas which need further research. This will enable the production of design guidelines for household and community scale UV water treatment, so that the WASH community will be able to take full advantage of the recent developments and standardizations in UV disinfection technology.

## Introduction

In 2016, WASH-related helminth infections (e.g. schistosomiasis, soil-transmitted helminthiases, taeniasis) were responsible for over 9.5 million years lost due to ill-health, disability or early death [1]. They are transmitted through contact with (or consumption of) water, food, and soil that contain the human infective stages of the parasite. Current control methods for combating these neglected tropical diseases (NTDs) are primarily focused on preventative chemotherapy with anthelmintic drugs, which has been effective at reducing the global health burden [2, 3]. However, reinfection is common and it is now widely recognized that an inter-sectoral approach is required for combatting many of these diseases [4–7]. In 2015 the World Health Organization (WHO) published their global strategy for WASH for NTDs, confirming that whilst WASH was one of the five key interventions in the global NTD roadmap published in 2012, little progress has been made in linking WASH and NTD programs [8]. More recently the WHO published “WASH and Health working together”, a toolkit for WASH and NTD programs based on the BEST (Behavior, Environment, Social inclusion, Treatment and care) framework [9]. Access to sanitation and clean water, and promotion of safe water practices are key interventions under the behavior and environmental components of the framework for many of the NTDs, including six helminthiases. Yet 29% of the global population do not have access to managed water supplies and 61% lack access to sanitation services [10]. Whilst piped water requires significant developments in regional infrastructure, household and community scale water treatment processes can be used to treat water collected from contaminated water bodies. This reduces exposure to helminth eggs and larvae by providing safe alternative water supplies for contact activities such as hand washing, bathing, and laundry.

UV disinfection is widely used for water and wastewater treatment in many parts of North America, Asia, and Europe. It has the benefits of forming no trihalomethanes or haloacetic acids, regulated by-products of chlorination, and can be successfully used against chlorine resistant pathogens such as *Cryptosporidium parvum* and *Giardia lamblia* [11, 12]. UV radiation is the part of the electromagnetic spectrum between 100 and 400 nm, which can be categorized into four types: UV-A (400–315 nm), UV-B (315–280 nm), UV-C or the germicidal range (280–200 nm), and Vacuum UV (200–100 nm). Unlike chlorination, UV disinfection does not necessarily kill pathogens. When a microorganism is exposed to UV light, most of the photons pass through it but some are absorbed by various cellular components. In the germicidal range, proteins and the nucleotide bases that make up DNA and RNA account for most of the absorption. Absorption by proteins is highest below 230 nm, but in this range water also strongly absorbs UV light, and high fluences are generally required for protein damage to occur. Lower fluences are required for absorption by DNA or RNA, which peaks at about 260 nm. All nucleotide bases absorb UV light, but absorption by the pyrimidine base thymine is the most critical for UV inactivation of microorganisms. When two thymine bases are adjacent to each other on a DNA chain, the absorption of a photon by one of the bases leads to a new chemical bond with the neighbouring thymine base, known as a dimer. In viruses that only contain RNA a similar reaction occurs between neighbouring uracil bases. The dimer changes the structure of the DNA or RNA and prevents the formation of new chains during replication, thereby inactivating the pathogen [13].

Conventional UV technologies use low pressure mercury filled arc lamps which produce near-monochromatic light at 253.7 nm, very close to the absorption maximum of DNA [13]. However, these lamps are made of fragile quartz and contain toxic mercury, which requires specialist handling and disposal. They also require relatively high input power and a reliable AC electricity supply. As a result, UV disinfection is often seen as incompatible with small scale water treatment in low income regions. The recent rapid development of UV-C LEDs offers an alternative source of UV light that may be more suitable for this context. UV-C LEDs are mercury free, durable, have a lower drive voltage than conventional mercury lamps, and can be powered by battery or photovoltaic supplies, so they can be used in rural or remote settings. UV-C LEDs are also much smaller than mercury lamps, which allows for novel design of water treatment systems, particularly point-of-use applications [14, 15]. The optical power of UV-C LEDs is currently relatively low, meaning devices need to be run for long periods of time to achieve sufficient inactivation of pathogens. The best wall plug efficiency (WPE, ratio of optical output power to electric input power) for a commercially available UV-C LED device is currently 4.1%, compared to 30 to 40% for low pressure mercury lamps [16, 17]. However, efficiency is improving and the WPE of commercial UV-C LED devices is expected to exceed 10% by 2021 [16]. Furthermore, in the last 15 years the cost of commercially available UV-C LEDs has decreased from over 1000 USD/mW to less than 1 USD/mW [18]. If these trends continue, and UV-C LED technology follows the path of visible LEDs, household and community scale UV disinfection of water may become a realistic option for low-income regions.

Sunlight is an alternative source of UV light and SODIS is now widely recognized as a sustainable form of small scale, e.g. household level, drinking water treatment. SODIS typically involves filling 2-liter polyethylene-terephthalate (PET) drinks bottles with water and placing them on a reflective surface for a minimum of six hours in direct sunlight (24 hours on overcast days). Pathogens are inactivated through a combination of heating and UV-A and UV-B disinfection [19].

Conventional UV disinfection and SODIS have been shown to be effective against a wide range of waterborne pathogens, but there is no guidance for their use against helminths, even though many can be spread through water. The aim of this research is to review existing literature on the UV sensitivity of WASH-related helminths, determine which helminths are more susceptible to this form of water treatment, and identify gaps in research which will inform future studies regarding the proper use of UV and SODIS for minimizing the spread of these diseases via water in low-income regions.

## Methods

This systematic review follows the guidelines of the Preferred Reporting Items for Systematic reviews and Meta-Analyses (PRISMA) [20]. The search took place between 1^st^ and 6^th^ June 2018 and included five databases: Web of Science, PubMed, The British Library, Scopus, and Google Scholar. All languages and document types were included, and databases were searched from inception to present day. The databases were searched for any combination of species name (Table 1) and common UV disinfection terms (UV, Ultra-violet, Ultraviolet, SODIS, Sunlight, Solar Disinfection) in the title. The search was not limited to NTDs; it included all WASH-related helminths that can infect humans (including zoonotic species) as listed on the Centre for Disease Control index of Parasites of Public Health Concern [21]. This includes species that are not necessarily waterborne but that can be transmitted through water if they enter water or wastewater (such as Soil-Transmitted Helminths which are spread through faeces). Class names cestode, nematode, and trematode, and common names, such as hookworm, were also included in the search. An example search strategy can be found in S1 Supporting Information.

**Table 1:** List of species included in the database search, grouped by class.

Cestodes	Nematodes	Trematodes
*Diphyllobothrium latum* *Diphyllobothrium pacificum* *Diphyllobothrium cordatum* *Diphyllobothrium ursi* *Diphyllobothrium dendriticum* *Diphyllobothrium lanceolatum* *Diphyllobothrium dalliae* *Diphyllobothrium yonagoensis* *Spirometra mansonoides* *Spirometra erinacei* *Spirometra mansoni* *Spirometra ranarum* *Echinococcus granulosus* *Echinococcus multilocularis* *Echinococcus vogeli* *Echinococcus oligarthrus* *Hymenolepis nana* *Taenia solium* *Taenia saginata* *Taenia asiatica*	*Ancylostoma brazilense* *Ancylostoma caninum* *Ancylostoma ceylanicum* *Ancylostoma duodenale* *Necator americanus* *Uncinaria stenocephala* *Angiostrongylus cantonensis* *Angiostrongylus costaricensi* *Parastrongylus costaricensis* *Ascaris lumbricoide* *Ascaris suum* *Anisakis simplex* *Pseudoterranova decipiens* *Capillaria hepatica* *Capillaria philippinensis* *Capillaria aerophila* *Dracunculus medinensis* *Gnathostoma spinigerum* *Gnathostoma hispidum* *Trichuris trichiura*	*Clonorchis sinensis* *Fasciola hepatica* *Fasciola gigantica* *Fasciolopsis buski* *Heterophyes* *Metagonimus yokogawai* *Opisthorchis viverrini* *Opisthorchis felineus* *Paragonimus westermani* *Schistosoma mansoni* *Schistosoma haematobium* *Schistosoma japonicum*

### Classification criteria

**Fig 1:**
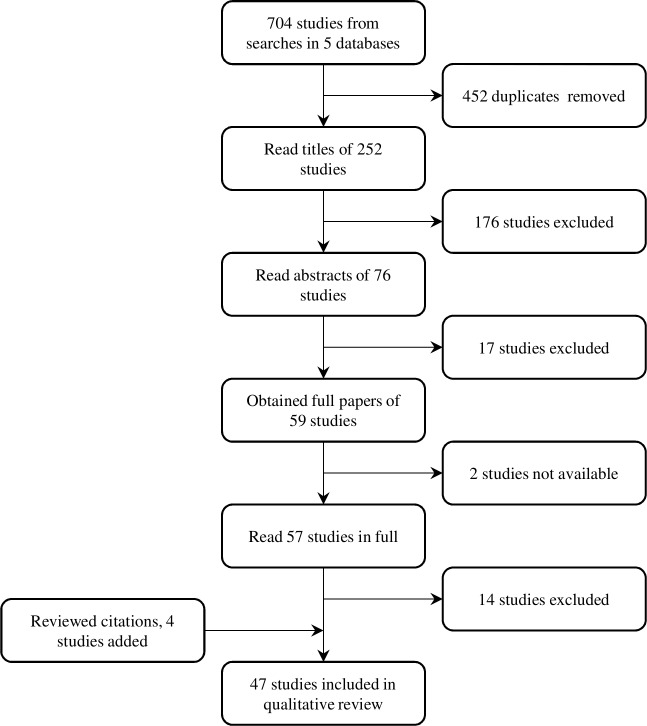
Classification flow diagram.

The studies were reviewed and classified according to the process flow diagram shown in Fig 1. First duplicates were removed and assigned code 1, then the papers were classified by title. Titles which suggested the studies were not about a relevant species or UV disinfection were removed and assigned codes 2–4 (animal species of the listed genera were included). The remaining abstracts were read and those that were about a non-waterborne life stage or primarily about the host response to a UV-attenuated vaccine were assigned codes 5 and 6, and removed. Papers for the remaining studies were obtained and read in full. Studies that provided limited information about the effect of UV light on the helminth or that contained significant errors (such as using a wavelength outside the UV range) were assigned codes 7 and 8 and were excluded; the remainder (code 9) were included in the review. Additional studies that were referenced in the papers in a way that suggested they were relevant to this review were also obtained, read in full, and assigned the relevant code.

Papers were obtained from Imperial College London Library, The British Library, and The Wellcome Trust and were read independently by the first and second authors. Papers that were not written in English were either translated using online translation software (Google Docs translation tool) or by Imperial College London students who were native speakers of the language. Notes were made on the studies and relevant information was extracted and included in a table (S1 Table). Any discrepancies between the first and second authors about which studies should be included were discussed and resolved.

### Data extraction

Where possible, the log reduction was calculated using the inactivation data presented in the studies and the equation $Log\;Reduction=-{log}_{10}\left(\frac{N}{N_{0}}\right)$, where *N* = proportion of viable organisms in the experimental sample and *N*_0_ = proportion of viable organisms in the control sample. If the survival percentage of the control sample was not stated in a paper, it was assumed that 100% survived (except for studies assessing the worm burden). If 100% of the experimental sample was inactivated, it was assumed that one organism survived in order to calculate the minimum log reduction; if the study only reported the percentage of organisms, then it was assumed that 1% survived. The log reduction values were then interpolated to calculate the UV fluence required to achieve a 1-log and 2-log reduction. If a 1-log reduction was not achieved in a study, then the data were extrapolated.

## Results and discussion

In total 704 papers were returned by the search, resulting in 252 individual studies, once duplicates were removed. After classifying the papers by title and abstract, 59 studies were selected to be read in full, but two were unavailable. 43 studies from the search were ultimately included in the review and four additional studies that were referenced in the original papers were added, resulting in a total of 47 studies.

### Species

Whilst 52 species of 23 genera were included in the search, results were returned for only 18 species of 10 genera: *Ancylostoma spp*, *Angiostrongylus spp*, *Ascaris spp*, *Echinococcus spp*, *Fasciola spp*, *Hymenolepis spp*, *Opisthorchis spp*, *Schistosoma spp*, *Taenia spp*, and *Trichuris spp* (Table 2).

**Table 2:** Summary of the genera included in this review [21]. One study [60] examined two genera, *Trichuris spp* and *Ascaris spp*, and is therefore listed twice.

Genus	NTD	# studies	Waterborne life stage	Transmission Route	Prevention
*Ancylostoma*	Soil-Transmitted Helminthiasis	3	Eggs	Skin contact with soil containing larvae.	Avoid walking bare foot. Reduce open defecation, effective sewage disposal.
*Angiostrongylus*	-	1	Third stage larvae	Food or water containing larvae including uncooked snails, slugs, or mollusk secretions.	Mainly food hygiene. Protect water from molluscs.
*Ascaris*	Soil-Transmitted Helminthiasis	16	Eggs	Food, water, or on hands contaminated with eggs passed in faeces.	Mainly handwashing and food hygiene. Reduce open defecation, effective sewage disposal.
*Echinococcus*	Echinococcosis	1	Eggs	Food, water, or on hands contaminated with eggs passed in dog faeces.	Mainly handwashing and food hygiene. Avoiding consumption of contaminated water.
*Fasciola*	Foodborne Trematodiases	1	Miracidia, cercariae, metacercariae	Food or water contaminated with metacercariae.	Avoid consumption of raw water plants and contaminated water.
*Hymenolepis*	-	1	Eggs	Food, water, or on hands contaminated with eggs passed in faeces.	Mainly handwashing. Avoid consumption of contaminated food and water. Reduce open defecation, effective sewage disposal.
*Opisthorchis*	Foodborne Trematodiases	1	Eggs, cercariae	Food containing metacercariae including undercooked fish.	Mainly food hygiene. Reduce open defecation, effective sewage disposal.
*Schistosoma*	Schistosomiasis	22	Miracidia, cercariae	Skin contact with water containing cercariae.	Mainly avoid contact with contaminated water. Reduce open defection, effective sewage disposal.
*Taenia*	Taeniasis	1	Eggs, gravid proglottids	Food containing cysticeri including undercooked pork and beef.	Mainly food hygiene. Reduce open defecation, effective sewage disposal.
*Trichuris*	Soil-Transmitted Helminthiasis	1	Eggs	Food, water, or on hands contaminated with eggs passed in faeces.	Mainly handwashing and food hygiene. Reduce open defecation, effective sewage disposal.

### UV light sources and experimental methods

Most studies used low pressure mercury arc lamps but other sources include: sunlight, solar simulators, fluorescent lamps emitting in the UV-A and UV-B range, medium pressure mercury lamps emitting over a broad spectrum in the UV-C range, and monochromatic excimer lamps emitting in the UV-C range. It is difficult to directly compare studies that used sunlight or simulated sunlight with mercury lamps, as sunlight contains almost no radiation in the UV-C (germicidal) range. Studies using sunlight and long wavelength sources (UV-A and UV-B) have therefore been reviewed separately to studies using UV-C sources. Where the source or wavelength was not stated in a paper it has been reviewed alongside the UV-C studies, as these are the most common.

The amount of UV light applied to a water sample is known as the fluence (mJ/cm^2^), which is a product of the exposure time (s) and fluence rate (mW/cm^2^). The protocol for calculating the fluence from low pressure mercury arc lamps in laboratory experiments was standardized only in 2003, using a bench top collimated beam apparatus. The method involves applying a series of corrections to the irradiance measured by a radiometer at the center of the beam, to account for reflection of light from the water surface, variation in irradiance over the surface area of the liquid, absorption of UV by the water column, and divergence of the “quasi-collimated” beam. Application of these factors to the measured irradiance will give the average germicidal fluence rate in the water sample. The method also requires that mercury lamps are allowed to warm up for at least 10 minutes to allow the output to stabilize and samples must be stirred during exposures to ensure all microorganisms receive the same fluence [22]. Only one study used this method to calculate the fluence, therefore the fluences stated for all the other studies should be considered approximate. Some studies only recorded the exposure time and the fluence could not be calculated.

Exposures were carried out in a number of different containers and the sample depth also varied between studies, from droplets on a glass cover slip to 25 mm deep samples in a culture dish [23, 24]. Different water matrices were also used for the exposures, for example deionized water, salt solutions, and filtered wastewater treatment plant effluent [25–32]. The sample depth and water matrix can affect the amount of UV light that is absorbed by the water; if this is not accounted for in the fluence calculation it may result in an overestimation of the average fluence in the sample. In the case of samples being exposed in very small volumes of water (e.g. droplets on coverslips), this may cause the samples to dry out, and it is difficult to separate the effect of drying from the effect of UV light on the inactivation of the target organism. Similarly, some UV sources are known to produce a considerable amount of heat, and not all experiments controlled the temperature of the samples, which may have also contributed to inactivation of the target organism.

Many microorganisms (e.g. bacteria) have the ability to reverse the damage caused by UV light through photoreactivation, dark repair, or excision repair [13]. However, the repair potential of helminths was explicitly examined in only one study and not all studies kept samples in the dark after UV exposure.

A variety of methods were used to determine the viability of helminths following exposure to UV light, the most common was to assess the ability of eggs or larvae to reach the next stage of development inside an animal host (also known as *in vivo* methods). *In vitro* methods such as assessing the motility or morphology of larvae, and the ability of eggs to embryonate in culture dishes, were also used. The most appropriate method may vary between genera. The *in vivo* method is often seen as the most definitive way to establish viability although it may not always produce the most reliable fluence-response curves. This is because helminths have complex lifecycles and often the number of organisms collected from a host is not directly proportional to the number of organisms in the inoculant. For example, in one study there was a considerable difference in the number of mice that developed infections depending on whether they were inoculated with 500 or 2,000 eggs (0% and 75%, respectively) and in another the number of control organisms recovered from the host varied notably between experiments (4–30%), possibly as a result of incomplete recovery or because of an unknown underlying issue which caused a reduction or increase in the intensity of infection in some of the host animals [33, 34]. Using the *in vivo* method is also likely to result in a higher level of inactivation than if an *in vitro* method is used to assess viability. This is because UV light inactivates pathogens by altering nucleic acids, and not all damage is immediately evident but can show up later in the development of the organism, which has been demonstrated in a number of studies in this review [24, 35–40]. Furthermore, as migration of eggs and larvae through the body can still pose a health risk, some authors suggest it is preferable to prevent entry to the bloodstream of the host and that it is necessary to demonstrate the organisms have been inactivated *in vitro* [41].

It is difficult to compare results between studies that use *in vivo* and *in vitro* methods. As the infection mechanism varies between genera, *in vitro* viability assessments may allow for better comparison, however further research is required to establish standardized methods for *in vitro* viability assessments of helminths. Selective dyes which stain only alive or dead cells may be suitable for this purpose and have previously been used to determine the viability of schistosome schistosomula [42]. One study in this review used methylene blue to identify dead schistosome cercariae which were stained violet blue, whilst live cercariae were left colorless [43].

### Sunlight, solar simulators, and long wavelength artificial sources

Twelve studies investigated the effect of sunlight and long wavelength UV light (313 and 390 nm) on seven species of helminth: *Ancylostoma caninum*, *Ancylostoma ceylanicum*, *Angiostrongylus cantonensis*, *Ascaris suum*, *Ascaris lumbricoides*, *Schistosoma mansoni*, and *Schistosoma haematobium*. In sunlight experiments the eggs or larvae were exposed for a minimum of 15 minutes to over six hours of continuous sunlight. Some studies also investigated the effect of exposure to intermittent sunlight over a period of days. Much shorter time periods were required when using artificial UV-A and UV-B sources.

*S*. *mansoni* cercariae were the most sensitive to natural sunlight, requiring 60 minutes for all cercariae to be rendered motionless in one study, even on cloudy days [44]. Prah and James found *S*. *mansoni* and *S*. *haematobium* miracidia were equally sensitive to sunlight, however longer exposures were required than in studies using cercariae [44, 45]. This suggests sensitivity to UV light may vary between different life-stages of the same species. Similarly, Spindler found single cell *A*. *suum* were more sensitive to sunlight than embryonated eggs [46] (Table 3). *A*. *suum* was the most resistant to sunlight; in one study single cell eggs were exposed to simulated sunlight between 290 and 800 nm at a fluence rate of 55 mW/cm^2^ for over six hours and only a 1.42-log reduction was achieved. However, it must be noted that a fluence rate over such a broad spectrum cannot be directly compared to a fluence rate in the UV range, as not all wavelengths have the same germicidal effectiveness. Furthermore, a high concentration of approximately 1 million eggs/mL was used and the study did not consider the effect of shielding, where organisms higher in the water column may protect lower ones from UV exposure [27]. The results of this study should therefore be considered conservative (i.e. under-estimate the true sensitivity of the eggs to UV light). Jones and Hollaender investigated the effect of simulated sunlight on *A*. *lumbricoides*, using a mercury source lamp which emitted light between 350 and 490 nm at a fluence rate of 0.1 to 30 mW/cm^2^. In this experiment the highest inactivation achieved was a 0.98-log reduction, but the authors noted that they would expect natural sunlight to be more damaging due to the presence of infrared radiation and higher temperatures. The samples were not mixed during the exposures; an effort was made to expose the eggs in single layers but there was an issue of “clumping” in some of the experiments [47].

**Table 3:** Sensitivity of some species to sunlight (interpolated data). Not all studies are shown because some contained insufficient information to calculate the log reduction.

Species	Conditions	Average exposure time (mins) to inactivate	
		1-log	2-log
*Ancylostoma caninum* (larvae) [51]	Direct sunlight, Korea	134	180
*Schistosoma haematobium* (miracidia) [45]	Consistent sunshine, UK	196	*-*
*Ascaris suum* (single cell) [46]	Direct sunlight, Puerto Rico	91	125
*Ascaris suum* (embryonated) [46]	Direct sunlight, Puerto Rico	300	311
*Ascaris suum* (single cell) [27]	Solar simulator 55 mW/cm^2^	317	-

Two studies investigated the effect of intermittent sunlight on *A*. *lumbricoides*; both found that eggs were able to survive for much longer periods (up to 60 hours) than in other studies that used continuous sunlight [27, 46, 48–50]. However, it should be noted that these experiments were carried out in Russia (at a high latitude) whereas other studies either used solar simulators or tropical sunlight containing higher levels of UV radiation (high fluence rates), which increases with proximity to the equator. Nenow confirmed that the germicidal effect of sunlight varies with altitude, suggesting that SODIS may be a more effective form of disinfection in communities located at higher altitudes, as shorter exposure times are required [50].

Of the hookworm species, no *A*. *caninum* larvae were able to survive 180 minutes exposure to sunlight [51], and only 60 seconds exposure to UV-A (390 nm) radiation was required for larvae of *A*. *ceylanicum* to become visibly sluggish. After 30 minutes exposure to UV-A light, larvae began to lose motility completely and when hamsters were orally infected with a dose of 100 larvae, no worms were able to develop [28] (Table 4). Similarly, larvae of the nematode *A*. *cantonensis* exposed for 15 minutes to UV-A light were unable to develop inside an animal host [52]. Only one study used UV-B light, which was shown to be relatively effective against *S*. *mansoni* miracidia. There were limited details on how the fluence was measured, but 86.1 mJ/cm^2^ (approximately 2 minutes 30 seconds) was sufficient to achieve a 2-log reduction in the number of daughter sporocysts in snails, even though the miracidia did not appear harmed. When exposed to fluorescent white light, immediately after irradiation, the miracidia were able to photoreactivate, with significantly higher numbers of sporocysts than in snails that were kept in the dark [23].

**Table 4:** Sensitivity of some species to long wavelength UV radiation (interpolated data). Not all studies are shown because some contained insufficient information to calculate the log reduction.

Species	Fluence Rate (mW/cm^2^)	Average exposure time (mins) to inactivate	
		1-log	2-log
*Schistosoma mansoni* (miracidia) [23]	0.578	<2	<3
*Angiostrongylus cantonensis* (larvae) [52]	-	4	-
*Ancylostoma ceylanicum* (larvae) [28]	-	9	-

The eggs and larvae in these experiments were exposed to sunlight or long wavelength UV in small amounts of water, with no more than 3 mL used in any of the experiments. However, SODIS is generally carried out in 2 L bottles, which are laid on their side and left in direct sunlight. The depth of the water column will therefore be much higher than in the studies included in this review, increasing the amount of UV light that is absorbed by the water. It is therefore recommended that SODIS experiments are also carried out in the containers that will be used by households and local communities.

SODIS is currently mainly used for drinking water treatment, and 2 L bottles are therefore appropriate reactors as the treated water can be drunk straight from the bottle. However, some helminthiases can be transmitted through poor personal hygiene and through contact activities such as bathing and laundry which require larger amounts of water and alternative reactors maybe required to effectively treat these volumes. Previous studies have used transparent plastic bags of various sizes as effective SODIS reactors although they have not been tested against helminths [53]. These have the advantage of a higher surface area to depth ratio whilst also allowing a larger volume of water to be treated. The underside of the bag can be coated with a reflective surface to increase the reflection of UV light into the water and they are cheap and easy to transport [54]. As the bags can be made to any size they may be more suitable for treating water for contact activities, however more research is required in this area.

### UV-C artificial sources

Germicidal mercury lamps (253.7 nm unless stated otherwise) were used in 24 studies and an additional 12 studies used other UV-C light sources or did not specify the wavelength. There was a very large range in the inactivation data for *A*. *suum* and *A*. *lumbricoides*, with fluences from 11 to 3,367 mJ/cm^2^ required to achieve a 1-log reduction (Table 5). Only one study by Brownell and Nelson used the industry standard protocol to evaluate the fluence though Lucio-Forster *et al*. applied some factors, correcting for reflection, absorption, and divergence of the UV beam. The results of these two studies are reasonably similar with fluences of 100 and 84 mJ/cm^2^ required to achieve 1-log inactivation of intact single cell eggs, respectively, although the difference is greater for 2-log inactivation [41, 55].

**Table 5:** Sensitivity of some species to UV-C radiation (interpolated data). Not all studies are shown because some contained insufficient information to calculate the log reduction.

**Species**	**Average fluence (mJ/cm**^**2**^**) to inactivate**	
	**1-log**	**2-log**
*Schistosoma japonicum* (cercariae) [26]	5	11
*Schistosoma mansoni* (cercariae) [63]	6	9
*Schistosoma mansoni* (cercariae) [64]	7	-
*Schistosoma japonicum* (cercariae) [62]	8	16
*Schistosoma mansoni* (cercariae) [65]	9	-
*Schistosoma japonicum* (cercariae) [68]	10	19
*Schistosoma japonicum* (cercariae) [25]	11	21
*Schistosoma japonicum* (cercariae) [67]	14	27
*Opisthorchis felineus* (eggs) [32]	27	-
*Ascaris suum* (embryonated eggs) [24]	11	22
*Ascaris lumbricoides* (single cell eggs) [31]	>26	-
*Ascaris suum* (single cell, decorticated) [41]	30	56
*Ascaris suum* (single cell eggs) [55]	84	168
*Ascaris suum* (single cell eggs) [41]	100	328
*Ascaris lumbricoides* (single cell eggs) [59]	3367	4748
*Taenia taeniaeformis* (eggs, decorticated) [34]	10	20
*Taenia taeniaeformis* (eggs) [34]	872	1300
**Species**	**Average time (mins) to inactivate**	
	**1-log**	**2-log**
*Schistosoma mansoni* (cercariae) [39]	0.46	-
*Schistosoma mansoni* (cercariae) [38]	0.96	-
*Schistosoma mansoni* (cercariae) [69]	6.00	8.10
*Fasciola gigantica* (miracidia) [61]	1.69	-
*Ancylostoma caninum* (larvae) [36]	11.78	-
*Hymenolepis diminuta* (eggs) [29]	16.00	-

A study by Tromba suggested *A*. *suum* is more sensitive to UV light, achieving a 2.21-log reduction at 24 mJ/cm^2^ [24], even though the eggs used were in a later stage of development and other studies reported that resistance to UV light increases with development stage [46, 50, 56]. However, Tromba determined viability of the eggs by assessing the worm burden in animal hosts, which may have resulted in a higher level of inactivation than if an *in vitro* method was used, as not all damage is immediately evident [24, 41, 57, 58]. Peng *et al*. found that deformities began to show two weeks after single cell eggs were exposed to UV light (unknown wavelength) for 10–20 minutes, even though during the first week of incubation development of irradiated eggs matched that of the controls. Eggs that were exposed for less than 10 minutes appeared to develop normally for longer periods, with deformities showing only after three weeks [35].

0.77-log reduction of *A*. *lumbric*oides was achieved at 20.3 mJ/cm^2^ using a prototype flow-through reactor, but in this study eggs were dissected from worm uteruses rather than collected from faeces or isolated from host intestines [31]. It is possible that these eggs were more sensitive to UV light because the eggshells may not have fully developed [41]. Furthermore, there was no information on how the fluence was calculated for the flow-through reactor.

One study compared the use of UV light and microwave radiation for disinfection of soil containing *A*. *lumbricoides* eggs, although experiments were also carried out in water. The authors found fluences over 3,000 mJ/cm^2^ were required to achieve any significant inactivation in water. Whilst some factors were applied during the fluence calculation, the collimating tube used in the experiments was 6 cm in diameter but only 10 cm long [59]. In this region the beam from mercury lamps is divergent, and radiometers can produce errors if they are used to measure the irradiance very close to the source. It is generally recommended that a collimating tube four times as long as the diameter is used with mercury sources [22]. It is therefore possible that the fluence was actually less than was stated in the paper.

Another study suggested that exposure to UV light actively increased the larval development of *A*. *lumbricoides*, even when exposed to fluences greater than 15,000 mJ/cm^2^ [30]. This contradicts the results of all the other studies included in this review and goes against the general understanding of the effect of UV light on microorganisms and UV disinfection. As with the previous study the irradiance was measured only 5 cm from the source and it is unclear if a collimating tube was used at all. The number of eggs in each sample was not specified and it is unclear if the suspensions were stirred. Furthermore, eggs were suspended in filtered secondary wastewater effluence, which may have absorbed a considerable amount of UV light, for which there was no correction applied. However, some inactivation would still have been expected. It is possible that some repair occurred (it is unclear if the samples were kept in the dark following exposure) or the accelerated development may be a result of the increase in temperature, which was not measured during the experiments, and has previously been shown to increase the rate of development in *Ascaris spp* [30].

An early study by Nolf compared two species of soil-transmitted helminth, and found that *Trichuris trichiura* was less sensitive to UV light (unknown wavelength) than *A*. *lumbricoides* [60]. Non-standard units were used to measure the extent of the exposure to UV light which means this paper cannot be compared to other studies, and there have been no further studies using *Trichuris spp* support this. The hookworm *A*. *caninum* appeared to be the most sensitive soil-transmitted helminth studied but the fluence was not recorded in these experiments. The initial log reductions achieved were relatively low, only 0.38 after five minutes exposure, however exposed larvae were not able to survive for as long as controls. Larvae exposed for five minutes did not live more than five days, whereas 52% of larvae exposed for 30 seconds were able to survive five days or more [36]. Unlike *Ascaris spp* and *Trichuris spp*, *Ancylostoma spp* larvae hatch from the egg in the environment, not inside the host, possibly explaining why this species is more sensitive to UV light. No studies were carried out using *Ancylostoma spp* eggs.

*Taenia taeniaeformis* eggs were very resistant to UV light, requiring 720 mJ/cm^2^ to achieve a 0.65-log reduction in the number of cysts recovered from the host when compared to control eggs. However, only one study investigated *Taenia spp* and there are few details of how the fluence was calculated. Furthermore, there was a notable difference in the number of cysts recovered from the controls in each of the experiments (4–30%) and it is unclear why this occurred. In the same study only 30 mJ/cm^2^ was required for 3-log reduction when the embryophore had been removed, suggesting that as with the eggshell for *Ascaris spp*, the embryophore is key to *Taenia spp* resistance to UV light [34, 41, 55].

The importance of the eggshell is less clear for other helminths in this review due to the lack of studies. A flow-through reactor with excimer lamps at 222 and 282 nm achieved a 0.92-log reduction in *Opisthorchis felineus* eggs in wastewater at a fluence of 25 mJ/cm^2^, suggesting it is relatively sensitive to UV light. However, a very small sample size was used, and it is unclear how the fluence was determined. Only two experiment samples were tested and the number of eggs in the samples prior to UV exposure was not known, only one control sample was tested to calculate the log-reductions [32]. *Hymenolepis diminuta* ova that were exposed to UV light for a minimum of 30 minutes were unable to develop into cysts. At 15 minutes exposure one cyst was able to develop, though this was deformed [29]. No infections developed in mice injected with 500 exposed *Echinococcus granulosus* eggs that had been exposed to UV light (unknown wavelength) for 24 hours. Yet, when mice were injected with 2,000 exposed eggs, 75% were able to develop infections, although significantly less eggs were able to develop into cysts than in control mice (0.15% compared to 0.7%). This was probably due to the proportion of viable embryos in each of the doses [33].

Of the trematodes, only one study used miracidia of *Fasciola gigantica*. There was a significant reduction in cercariae shed from snails when they were infected with one exposed miracidium, compared to one control miracidium, even at very short exposure times less than 70 seconds [61]. The effect of UV light on S*chistosoma spp* has been the most widely studied and it is the most sensitive helminth in this review. The majority of papers were immunization studies, investigating the use of UV-attenuated cercariae to produce a vaccine against human schistosomiasis. In these experiments cercariae were exposed to a fluence high enough to cause damage and prevent development into adult worms, but that still allowed penetration of the host’s skin. The focus was therefore on the worm burden, and details of the exposure methods were often limited. In the immunization papers a 1-log reduction in worm burden was achieved with fluences of 5–14 mJ/cm^2^ [25, 26, 43, 62–68] or exposure times of less than one minute [39]. The direct effect of UV light on cercariae was first studied by Krakower in 1940 who found 45 minutes exposure to a mercury lamp (unknown wavelength) was required to kill the whole sample. Shorter exposures were still able to cause damage, making the cercariae less motile than the control samples, though they were able to recover from their injuries within 30 minutes and survived for as long as the controls, suggesting schistosome cercariae have some repair potential [44]. Standen and Fuller found that only four minutes was required to kill 100% of *S*. *mansoni* cercariae in their study, but the mercury lamp used was very near to the sample (2 cm) and it is unclear if the authors controlled the water temperature [69]. Older mercury lamps are known to have produced a lot of heat and cercariae are inactivated within minutes at 45°C and almost instantly at temperatures above 50°C [70].

Ghandour and Webbe studied the effect of UV light on the ability of *S*. *mansoni* and *S*. *haematobium* cercariae to penetrate skin. There was a significant increase in mortality during skin penetration when cercariae were exposed for 5–20 seconds, even though they did not appear to be harmed. 10–11% of exposed cercariae were unable to penetrate at all compared to 2–3% of the control sample [37, 38]. Another study found that short exposure times caused a reduction in the motility of cercariae, but this only became apparent four hours after exposure [39]. Cercariae penetrate skin through enzyme activity and mechanical action, a combination of motility reduction and inhibition of enzymes may have prevented cercarial penetration.

Two studies used scanning electron microscopy (SEM) to examine the physical damage caused by UV light to *S*. *mansoni*. Mohamed showed that adult worms developed from irradiated cercariae had lost their spikes and suffered from torn tubercles and lesions, causing sexual anomalies and sterility, possibly explaining the reduction in fecundity of worms derived from irradiated cercariae in other studies [39, 64, 71]. Later Dajem & Mostafa used SEM to examine the damage on the surface of cercariae and discovered that irradiated samples appeared to be physically the same as control cercariae, suggesting the damage observed in adult worms is either a result of mutagenic effects of UV light which only appear later in development, or as a result of the hosts immune response to irradiated cercariae [40]. Another study found that UV exposure modified the structure of molecules on the surface of *S*. *mansoni* cercariae, even though no morphological changes occurred. This may have caused an enhanced immune response by the host [72].

Significantly more male *S*. *mansoni* worms were able to develop from irradiated cercariae in one study, suggesting that males may be more resistant to UV light than females [73]. This also may explain the reduction in fecundity observed in other studies, but further research is required to confirm this [39, 64]. *S*. *mansoni* and *S*. *japonicum* were shown to be equally sensitive to UV light, suggesting the inactivation mechanism is the same in both species [66]. Only one study used *S*. *haematobium* cercariae, which were found to be slightly more resistant that *S*. *mansoni* cercariae, however no statistical analysis was performed [38]. Prah and James found there was no difference in the response of *S*. *mansoni* and *S*. *haematobium* miracidia to UV light from mercury source lamps. Experiments in this study were repeated with 1% turbid water, and a 15.4% reduction in the rate of movement of miracidia was observed, compared to a 60.3% reduction when distilled water was used [45]. However, it should also be noted that distilled water has been shown to kill schistosome cercariae, and it may have a similar effect on miracidia [74]. Whilst many different suspension media have been used, this was the only study in the review that investigated the impact of turbidity or the water matrix on UV disinfection. If UV or solar disinfection is to be used effectively for household and community scale water treatment this aspect requires further research, preferably using water samples collected from the environment. If water collected from local waterbodies is of particularly poor quality (e.g. high turbidity, iron, or organic matter content), consideration may need to be given to pre-treatment, such as filtration or sedimentation.

### Conclusion

With the recent introduction of UV-C LED technology into the water sector, UV disinfection could be a realistic option for sustainable water treatment in low-income regions in the near future, to provide safe water supplies for water contact activities such as bathing, laundry, and to improve hygiene. Compared to bacterial and viral pathogens there has been little research into the effectiveness of UV light at inactivating helminth eggs or larvae, which are endemic to many developing countries. The majority of studies in this review investigated the effect of UV light on either *Schistosoma spp* or *Ascaris spp*, and many were immunization studies used for developing UV-attenuated vaccines, with a focus on the host response to irradiated larvae or eggs, not complete inactivation of the target organism or applications to water treatment.

There were limitations to almost all of the studies, the most significant being the lack of a standardized procedure for calculating the UV fluence to which samples were exposed. 68% of studies were carried out before the industry standard protocol for fluence measurement was published in 2003 [22]. In the SODIS studies, experiments were carried out using very small amounts of water which is not representative of how disinfection will take place in practice. Very few studies considered the impact of water quality or accounted for the absorbance of UV by the water column or the effect of shielding caused by suspended particles and other organisms. Mercury lamps are known to produce considerable amounts of heat and it is not clear which of the studies controlled the water temperature. In some studies the fluence was not recorded at all and only one study investigated the repair potential. The methods for determining the viability of larvae or eggs varied, even between papers using the same genera, and this resulted in large ranges in the fluence response, most notably for *Ascaris spp*. Furthermore, the survival percentage of control samples was not stated in all studies and assumptions were made to calculate the log reductions presented in this review.

These limitations make it difficult to directly compare the studies, however some conclusions can be drawn. All helminths included in this review could be inactivated by UV light at certain fluences and wavelengths, but the number of species studied was limited. Helminths which hatch from the egg in the environment were generally more sensitive to UV light than species which stayed in the egg until after they had infected the host. Studies found that eggs were much more sensitive to UV when the shell or embryophore had been removed, suggesting they play a key role in the resistance to UV light for some species. Fluences in excess of 80 mJ/cm^2^ were required to achieve a 1-log inactivation of *Ascaris spp* and *Taenia spp* eggs, over twice the current minimum fluence required by some European countries for the treatment of publicly supplied drinking water [75, 76]. UV disinfection may therefore not be the most efficient form of water treatment for these helminths. UV disinfection may be particularly effective against *Schistosoma spp* which was consistently the most sensitive to UV light in this review, however further experimental research is required using the standard fluence measurement protocol.

This systematic review has demonstrated that evidence exists to suggest that UV disinfection is effective against some helminths, but the data covers a limited number of species and is insufficient to produce detailed recommendations for household or community scale UV or solar disinfection of water in endemic regions. To aid the design of these water treatment systems we recommend the following for future studies on UV disinfection of WASH-related helminths:

1. The industry standard Bolton & Linden protocol should be used to accurately determine the UV fluence in experiments using mercury lamps to ensure the results are comparable and repeatable, taking into consideration the potential for photoreactivation of the target organisms. There is currently no standard protocol for determining the UV fluence from UV-C LEDs. Until this has been agreed by the UV industry researchers should stay up-to-date with developments in this area and studies using these devices should include a detailed methods section, clearly stating the type of device and protocol used.
2. The methods for determining viability of helminths should be standardized, possibly with a move towards *in vitro* methods, which allow for better comparisons between genera and are not reliant on animal testing.
3. Solar disinfection (SODIS) experiments should be carried out in countries where the helminths are endemic, using appropriate containers and storage times, to determine if current SODIS guidelines are sufficient for helminth inactivation.
4. Experiments should be carried out in lab conditions to determine the fluence response relationship of the target organisms, and in field conditions to fully understand the effectiveness of the UV disinfection in real water matrices. Water samples and helminth eggs or larvae collected from the environment should be used and recommendations made regarding the turbidity and UV absorbance limits at which UV and solar disinfection can be used for helminth inactivation.
5. Helminths which hatch from their egg in the environment should be prioritized for UV disinfection studies, as larval forms appear to be more sensitive to UV light than helminth eggs.

## Supporting information

S1 Supporting Information Search protocol. Example database search strategy.
(DOCX)

S2 Supporting Information PRISMA 2009 checklist. (DOCX)

S1 Table Search results. Classification codes, a list of excluded and included studies, and extracted data.
(XLSX)

S2 Table Calculations. Calculations of log reductions and interpolation of data extracted from studies.
(XLSX)
