# Supplementary material for: Ultraviolet sensitivity of WASH (water, sanitation, and hygiene) -related helminths: A systematic review
Source: PLoS Negl Trop Dis. 2019 Sep 19;13(9):e0007777. doi: 10.1371/journal.pntd.0007777 (PMC6772140; doi:10.1371/journal.pntd.0007777)
Supplement: S1 Supporting Information — Example database search strategy. (DOCX) [file pntd.0007777.s001.docx]

**S1 Supporting Information. Search protocol.** Example database search strategy.

**Example search strategy (Web of Science)**

TI = (Ancylostom* OR brazilense OR caninum OR ceylanicum OR duodenale OR Necator OR americanus OR Uncinaria OR stenocephala OR Hookworm OR Angiostrongyl* OR cantonensis OR costaricensis OR Parastrongyl* OR Anisaki* OR simplex OR Pseudoterranova OR decipiens OR Ascari* OR lumbricoides OR suum OR Roundworm OR Capillaria* OR hepatica OR philippinensis OR aerophila OR Clonorchi* OR sinensis OR Diphyllobothri* OR latum OR pacificum OR cordatum OR ursi OR dendriticum OR lanceolatum OR dalliae OR yonagoensis OR Spirometra OR mansonoides OR erinacei OR ranarum OR Dracuncul* OR medinensis OR Guinea worm OR Echinococc* OR granulosus OR multilocularis OR vogeli OR oligarthrus OR Hydatid OR Fasciol* OR hepatica OR gigantica OR Fasciolopsi* OR buski OR Gnathostom* OR spinigerum OR hispidum OR Heterophy* OR heterophyes OR Metagonim* OR yokogawai OR Hymenolepi* OR nana OR Opisthorchi* OR viverrini OR felineus OR Paragonim* OR westermani OR Schistosom* OR mansoni OR haematobium OR japonicum OR Bilharz* OR Taenia OR solium OR saginata OR asiatica OR Tapeworm OR cysticercosis OR Trichuris OR trichiura OR Whipworm OR Helminth OR STH OR Trematode OR Cestode OR Nematode OR Acanthocephalan OR Flatworm OR Fluke) AND TI = (UV OR Ultra-violet OR Ultraviolet OR SODIS OR Sunlight OR Solar Disinfection) NOT TS=(herpes)

Note: Herpes was excluded from the search strategy as it produced a large number of irrelevant studies.
